# Technical feasibility of [^18^F]FET and [^18^F]FAZA PET guided radiotherapy in a F98 glioblastoma rat model

**DOI:** 10.1186/s13014-019-1290-4

**Published:** 2019-05-30

**Authors:** Jeroen Verhoeven, Julie Bolcaen, Valerie De Meulenaere, Ken Kersemans, Benedicte Descamps, Sam Donche, Caroline Van den Broecke, Tom Boterberg, Jean-Pierre Kalala, Karel Deblaere, Christian Vanhove, Filip De Vos, Ingeborg Goethals

**Affiliations:** 1 Laboratory of Radiopharmacy, Ghent University, Ghent, Belgium; 2 Ghent University Hospital, Department of Nuclear Medicine, Ghent, Belgium; 3 National Research Foundation (NRF), iThemba LABS, Somerset West, South Africa; 4 Ghent University Hospital, Department of Radiology and Medical Imaging, Ghent, Belgium; 5 IBiTech-MEDISIP Ghent University, Department of Electronics and Information Systems, Ghent, Belgium; 6 Ghent University Hospital, Department of Pathology, Ghent, Belgium; 7 Ghent University Hospital, Department of Radiation Oncology, Ghent, Belgium; 8 Ghent University Hospital, Department of Neurosurgery, Ghent, Belgium

**Keywords:** Glioblastoma, PET boosting, FET, FAZA, Radiotherapy, Preclinical

## Abstract

**Background:**

Glioblastoma (GB) is the most common primary malignant brain tumor. Standard medical treatment consists of a maximal safe surgical resection, subsequently radiation therapy (RT) and chemotherapy with temozolomide (TMZ). An accurate definition of the tumor volume is of utmost importance for guiding RT. In this project we investigated the feasibility and treatment response of subvolume boosting to a PET-defined tumor part.

**Method:**

F98 GB cells inoculated in the rat brain were imaged using T2- and contrast-enhanced T1-weighted (T1w) MRI. A dose of 20 Gy (5 × 5 mm^2^) was delivered to the target volume delineated based on T1w MRI for three treatment groups. Two of those treatment groups received an additional radiation boost of 5 Gy (1 × 1 mm^2^) delivered to the region either with maximum [^18^F]FET or [^18^F]FAZA PET tracer uptake, respectively. All therapy groups received intraperitoneal (IP) injections of TMZ. Finally, a control group received no RT and only control IP injections. The average, minimum and maximum dose, as well as the D_90_-, D_50_- and D_2_- values were calculated for nine rats using both RT plans. To evaluate response to therapy, follow-up tumor volumes were delineated based on T1w MRI.

**Results:**

When comparing the dose volume histograms, a significant difference was found exclusively between the D_2_-values. A significant difference in tumor growth was only found between active therapy and sham therapy respectively, while no significant differences were found when comparing the three treatment groups.

**Conclusion:**

In this study we showed the feasibility of PET guided subvolume boosting of F98 glioblastoma in rats. No evidence was found for a beneficial effect regarding tumor response. However, improvements for dose targeting in rodents and studies investigating new targeted drugs for GB treatment are mandatory.

**Electronic supplementary material:**

The online version of this article (10.1186/s13014-019-1290-4) contains supplementary material, which is available to authorized users.

## Background

Glioblastoma (GB) is the most common primary malignant brain tumor. It has been given the highest grade of WHO classification of glioma (WHO grade IV) and is a highly invasive solid tumor [1–3]. Histologically, GBs are characterized by increased vascularity, nuclear atypia, necrosis and increased mitotic activity. Tumor cell infiltration deep into the surrounding brain parenchyma renders a complete surgical resection elusive leading inevitably to tumor recurrence in most cases [4, 5].

Several factors such as lesion size, location and growth rate determine the clinical presentation of patients with GB. Headache, seizures, personality changes and focal neurological deficits are common symptoms of GB [1]. The clinical course of GB is rapidly progressive, with a mean survival time between six to fourteen months. Standard medical treatment consists preferably, and if possible, of a maximal safe surgical resection. Subsequently radiotherapy (RT; 60 Gy in 30 fractions) and concomitant chemotherapy with temozolomide (TMZ; 75 mg/m^2^ during six weeks) followed by adjuvant chemotherapy with TMZ (150–200 mg/m^2^ × 5 days, during 6 cycli of 28 days) are administered. Even with this treatment strategy, patients with GB typically develop fast post-operative progression resulting in fatal outcome for the majority of them [1, 6].

**Fig. 1:**
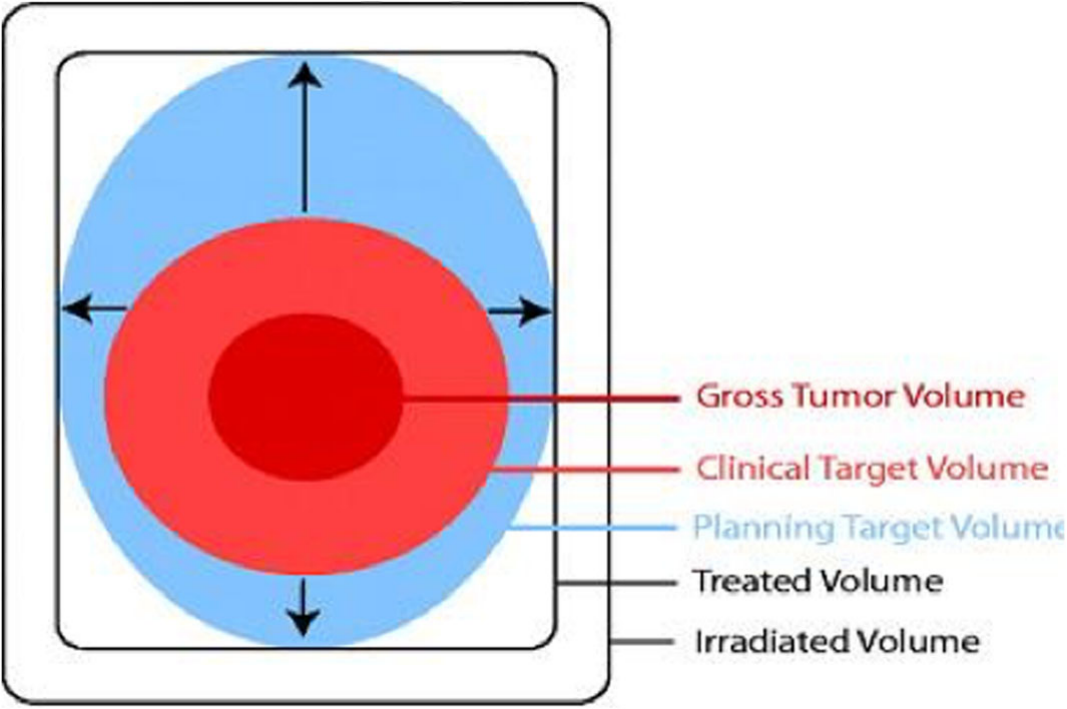
Schematic illustration of the different volumes as defined in ICRU Report 50. Gross tumor volume (GTV) denotes the demonstrated tumor. Clinical target volume (CTV) takes the tumor infiltration outside the GTV into account. The planning target volume (PTV) consists of the CTV and a margin to account for the interfractional and intrafractional variations [10]. Reproduced with permission of the International Commission on Radiation Units and Measurements, https://ICRU.org

To improve this poor survival rate, new treatments are crucial. RT is an essential part of (postoperative) GB treatment, as has been demonstrated in the past [7]. RT planning is currently based on contrast-enhanced computed tomography (CT) and magnetic resonance imaging (MRI). The International Commission on Radiation Units and Measurements Report No. 50 (ICRU-50) proposed to delineate three different target volumes based on diagnostic imaging (Fig. 1) [8]. The gross tumor volume (GTV) reflects the macroscopical tumor volume as assessed by contrast-enhanced anatomical imaging. To include the microscopic infiltration into the adjacent healthy brain tissue, an extra margin is proposed being the clinical target volume (CTV). Finally, the planning target volume (PTV) also accounts for interfractional and intrafractional variations [9, 10].

In 2000, Ling et al. (*10*) introduced the biological target volume (BTV). The BTV is based on the concept that irradiation of the tumor region with an adapted dose based on proliferative activity, could result in a better outcome. To include BTV in the treatment protocol, a non-uniform dose can be delivered to the target volume based on functional imaging (i.e. dose painting) [11, 12]. Hence, additional biological information from positron emission tomography (PET) may have added value for defining this RT target tumor volume [12, 13]. The use of BTV in a (pre)clinical set up has been evaluated by Trani et al. (*11*) where they demonstrate the technical feasibility of preclinical dose painting strategies. However, more preclinical studies that confirm these results, or are aimed at testing similar biological hypotheses, are needed [12, 14].

﻿O-(2-[^18^F]fluoroethyl)-L-tyrosine ([^18^F]FET) and ﻿1-α-D-(5-deoxy-5-[^18^F]-fluoroarabinofuranosyl)-2-nitroimidazole ([^18^F]FAZA) were selected as tracers for PET-guided RT. Uptake of the amino acid PET tracer, [^18^F]FET, correlates with tumor cell density and proliferation and is therefore able to identify tumor regions susceptible to relapse [15, 16]. Uptake of the hypoxia tracer, [^18^F]FAZA, is correlated with tumor hypoxia, which can cause radioresistance [16, 17]. As such, a higher radiation dose targeting those specific regions might be beneficial [18].

Rat models have remained the mainstay of neuro-oncology research for over 30 years. The C6 glioma has been used extensively for a variety of studies, however, it has the potential to evoke an immune response and it is not syngeneic to any inbred strain [19, 20]. The 9 L gliosarcoma has been widely used but can be immunogenic in syngeneic hosts. The U251 xenograft model shows histological characteristics of human GB and displays genetic similarities to human GB but is criticized for an inadequate tumor-host immune response [21]. The U87 GB model displays key dissimilarities to human GB at the histopathological level. Unlike GB, U87 tumors show a non-diffusely infiltrative growth pattern, with more homogeneous and leaky vessels [21, 22]. It is also worth mentioning that patient-derived glioblastoma models would necessitate the use of nude rats, which are much more expensive. Taking these models in consideration, the F98 GB model was selected for this preclinical study based on the presence of all the necessary histologic characteristics, the costs and the extensive experience of our research group with this model [23–26]. Recently, in 2017 Bolcaen et al. optimized a protocol for PET- and MRI-guided irradiation of a GB rat model using a micro-irradiator [23]. Using this approach, it is our goal to conduct a proof of concept for the application of PET-defined subvolume boosting using two different PET tracers and subsequently evaluate treatment outcome using MRI.

## Materials & methods

### F98 GB model

The study was approved by the Ghent University ethics committee for animal experiments (ECD 12/28-A2). All animals were kept and handled according to the European guidelines (2010/63/EU) and housed under environmentally controlled conditions (12 h normal light/dark cycles, 20 °C – 24 °C and 40–70% relative humidity) with food and water ad libitum.

The GB F98 rat model was developed as described by Bolcaen et al. [26]. In summary, F98 rat GB cells were cultured as monolayers for three weeks. 54 Female Fisher F344 rats (Charles River®) were anesthetized with ketamine/xylazine (i.p., 4/3; 0.13 mL/100 g), and immobilized using a stereotactic frame (Model 902 Dual Small Animal Stereotaxic frame, Kopf®). After shaving, the head was swabbed with betadine and the skull was exposed through a longitudinal 1 cm scalp incision. Using a diamond drill (Dremel®), a 1 mm hole was made through the skull (2.5 mm lateral to the bregma in the right frontal hemisphere). A stereotactically guided 1 mL insulin needle was inserted at a depth of 3 mm and 5 μL of cell suspension containing 20,000 F98 cells in phosphate-buffered saline was delivered. This cell suspension was injected using a microsyringe pumpcontroller (Micro 4TM, World Precision Instruments, Sarasota, USA) over a 2-min period. The syringe was slowly withdrawn 1 min post inoculation and the incision was closed with bone wax (Aesculap AG®) and sutured.

### MRI

Eight days post inoculation small animal MRI was performed on a 7 T system (PharmaScan 70/16, Bruker, Ettlingen, Germany) to confirm tumor growth. Rats were anesthetized with 2% isoflurane mixed with oxygen administered at a flow rate of 0.3 L/min. To enable the injection of a gadolinium containing contrast agent (Dotarem®, Guerbet, 2 mmol/kg), a 30-Gauge needle connected to a 60 cm long PE tube was placed intravenously in the lateral tail vein. A rat brain surface coil (Rapid Biomedical, Rimpar®, Germany) was applied around the head of the animal followed by positioning of the bed in a 72 mm whole body transmitter coil (Rapid Biomedical, Rimpar®, Germany). A T2-weighted scan (T2w SE RARE, TR/TE 3661/37.1 ms, 109 μm isotropic in plane resolution, 4 averages, TA 9′45″) was performed to localize the tumor. If tumor was visually confirmed, a 5 min T1w scan was obtained after injection of the gadolinium containing contrast agent.

### PET imaging

PET images were acquired on a small animal PET scanner (FLEX Triumph II, TriFoil Imaging®, Northridge, CA, USA). A 30 min PET scan was obtained with [^18^F]FET or [^18^F]FAZA (±37 MBq; 30 min or 2 h post-injection, respectively). This time frame allowed to acquire the MRI scans during tracer uptake. Reconstruction of the PET scans was done by a 2D Maximum Likelihood Expectation Maximization (MLEM) algorithm (LabPET Version 1.12.1, TriFoil Imaging, Northridge, CA, USA) applying 50 iterations and a using voxel size of 0.5 × 0.5 × 1.157 mm^3^ into 200 × 200 × 64 matrix.

### Treatment groups

**Fig. 2:**
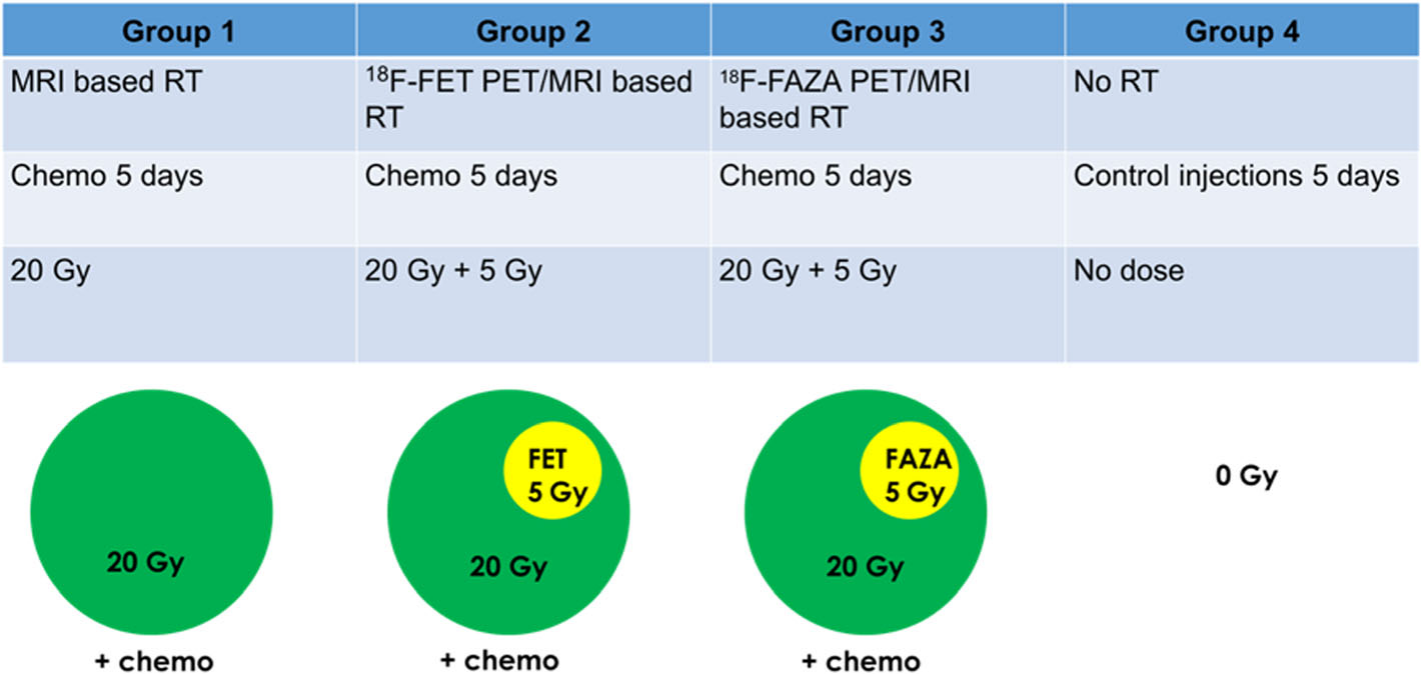
Overview of the four research groups. A dose of 20 Gy was delivered to the target volume delineated based on T1w MRI for three treatment groups. Two of those treatment groups received an additional radiation boost of 5 Gy delivered to the region either with maximum [^18^F]FET or [^18^F]FAZA PET tracer uptake, respectively. All therapy groups received intraperitoneal (IP) injections of TMZ. Finally, a control group received no RT and only control IP injections

Different therapy groups were defined based on the definition of the target volumes for brain tumor irradiation. For the MRI based RT group (*n* = 5), the isocenter was placed in the middle of the contrast enhancement on T1-weighted (T1w) MRI (Fig. 2). The [^18^F]FET-PET based RT (*n* = 6) and [^18^F]FAZA-PET based RT (*n* = 4) groups received an additional radiation boost of 5 Gy delivered to the region either with maximum [^18^F]FET or [^18^F]FAZA PET tracer uptake, respectively.

All therapy groups received concomitant treatment consisting of intraperitoneal (IP) injections of TMZ on five consecutive days, starting on the same day as the RT [27, 28]. Therefore, 29 mg/kg TMZ (Sigma-Aldrich®) was dissolved in saline with 25% dimethylsulfoxide.

Finally, a control group (n = 5) received no RT and only control IP injections with an equal amount of dimethylsulfoxide and saline on five consecutive days.

Animals with MRI-confirmed GB-tumor were randomly assigned to the abovementioned groups.

### Radiation treatment

Radiotherapy treatment was performed using a small animal radiation research platform (SARRP, Xstrahl®, Surrey, UK). MRI-based RT in the F98 GB rat model was already optimized by our research group [23, 29]. A gadolinium-based contrast agent was intravenously injected, followed by fixation of the animal on a multimodality bed. Two water filled capillaries were used as markers and were placed on the right side and in the middle of the rat head. After acquiring contrast-enhanced T1w spin-echo scans, the rat was transported to the four-axis robotic positioning table of the SARRP, while fixed on the multimodality bed. A treatment planning CT was obtained using the following acquisition parameters: 50 kV tube voltage, 600 μA tube current, 360 projection acquired over 360 degrees using a 1 mm Al-filter, resulting in a total acquisition time of 1 min. The acquired projection data were reconstructed using an isotropic voxel size of 0.2 mm. After importing the planning CT into the treatment planning software (Muriplan, Version 2.0.6, Xstrahl®, UK), manual segmentation was performed to distinguish air, soft tissue and bone. Co-registration with the MRI was done manually using the capillary markers and the skull. Based on the contrast-enhanced T1w MRI scan the isocenter of the radiation bundle was set in the middle of the tumor region. A single dose of 20 Gy was delivered by applying three non-coplanar arcs (120°) using a 5 × 5 mm^2^ collimator to include minor position changes of the rat head during execution of the treatment.

For the PET/MRI-based RT planning, co-registration of PET and planning CT was performed using PMOD (version 3.31, PMOD technologies, Ltd). First, the contrast-enhanced T1w MRI scan was co-registered with the planning CT as described above. Then, the PET image was co-registered with the planning CT. Finally, the coordinates of the isocenters based on 1) contrast enhancement on the T1w MRI scan and 2) based on maximum tracer uptake in the PET image were determined in PMOD and transferred to Muriplan. The isocenter indicated on the MRI scan received a single dose of 20 Gy using a 5 × 5 mm^2^ collimator as described above, while the isocenter based on maximum PET tracer uptake received an additional 5 Gy boost using a 1 × 1 mm^2^ collimator. The methodology has been described in detail in a recently published Jove Movie [23].

### Chemotherapy

Concomitant chemotherapy was given on five consecutive days starting on the same day as the RT. Therefore, IP injections of 29 mg/kg TMZ (Sigma-Aldrich®) dissolved in saline with 25% dimethylsulfoxide were performed. The control group received IP injections with an equal amount of dimethylsulfoxide and saline on five consecutive days [27, 28].

### Histological characterization

At the end of the experiment or when the humane endpoints were reached (> 20% weight loss, tumor volume > 40% of total brain volume based on MRI or signs of ataxia) rats were euthanized by an IV injection of pentobarbital (120 mg/kg). From five animals the brain was isolated, dissected, immersed in 4% paraformaldehyde for 24 h and embedded in paraffin. Then, the brain was partly sectioned into 5 μm slices and stained with hematoxylin and eosin (HE) in order to histologically characterize the tumor.

### Comparison of [^18^F]FET and [^18^F]FAZA PET

The uptake of both tracers was studied in the same F98 GB rat (*n* = 3). Additionally, the pre-RT PET scans of the [^18^F]FET-PET based RT (*n* = 6) and [^18^F]FAZA-PET (*n* = 4) based RT groups were analyzed. Using PMOD, rigid body transformations were applied to co-register the PET and MRI scans. Volumes of interest (VOI) were drawn manually and included the contrast-enhanced region on the T1w MRI scans. Cubic VOIs of 2 × 2 × 2 mm^3^ located in the contralateral region were used as a reference (background region). Tracer uptake in the VOI at each time frame was converted to a standardized uptake value (SUV) according to the following formula:

SUV = (Radioactivity in VOI expressed in Bq/ml / injected activity in Bq) x body weight in g.

Injected activity was corrected for radioactive decay and residual activity in the syringe. Standardized uptake values (SUV_mean_ and SUV_max_) and tumor-to-background ratios (TBR_mean_ and TBR_max_) were calculated.

### Dose volume histograms

To compare the dose of MRI-based RT with PET/MRI-based sub-volume boosting, dose volume histograms (DVH) were analyzed. Using Muriplan, the DVHs were determined within the overlapping volume of the three rotating 5 × 5 mm^2^ bundles using Muriplan. The average, maximum and minimum dose, as well as the D_2_-, D_50_-, and D_90_-values, were determined and compared between different RT treatment plans. D_x_ stands for the dose that x % of the tissue volume received.

### Assessment of therapy response

Tumor growth was evaluated by obtaining sequential MRI scans 2, 5, 9, 12, 14, and 16 days after initiating the treatment. Tumor volumes were measured by manually outlining the tumor on individual slices of contrast-enhanced T1w images using PMOD.

### Statistical analysis

Statistical analyses were done using SPSS software (SPSS Statistics 23 software). If applicable, a Mann-Whitney U test was applied. A probability value of *p* < 0.05 was considered statistically significant. Values are presented as mean ± SD.

## Results

### F98 GB rat model

**Fig. 3:**
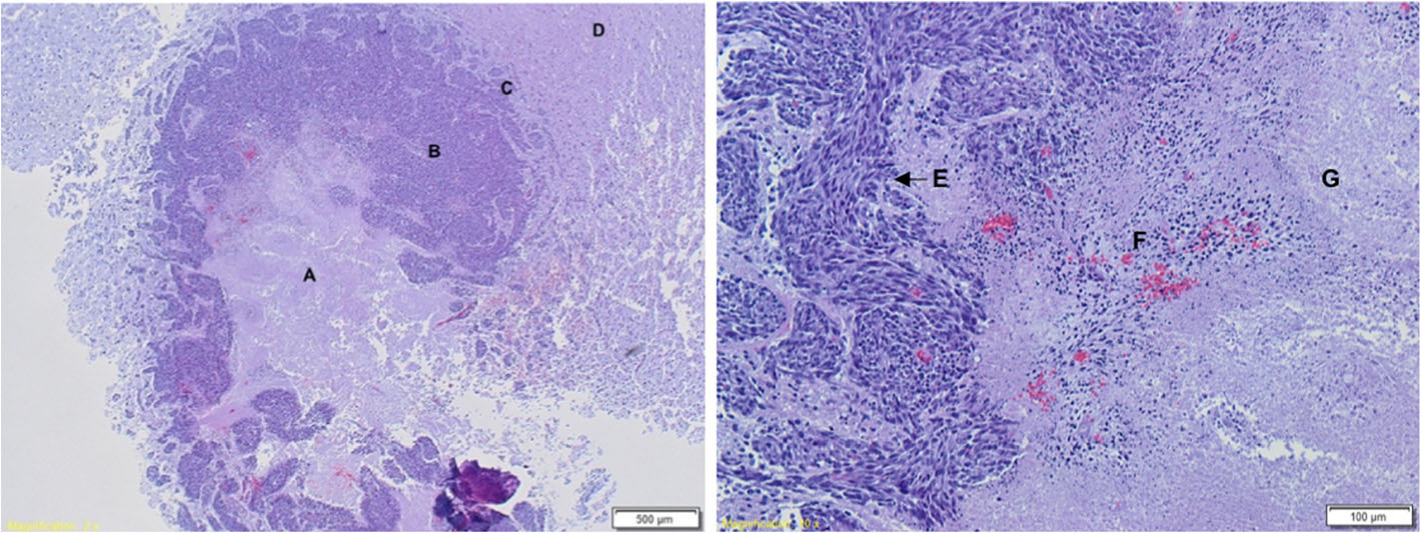
HE staining of rat 7 (MRI based RT group, 32 days post inoculation) confirms the presence of GB. On the overview picture (left) central tumor necrosis (**a**), tumor (**b**), infiltration (**c**) of the surrounding healthy brain tissue (**d**) can be seen. The detailed section shows increased cellularity, more nuclear pleomorphism and atypia, increased proliferation (**e**), microvascular hyperplasia (**f**) and necrosis (**g**)

HE staining of rat 7 (Fig. 3) shows typical characteristics of GB. On the overview picture (left) tumor necrosis (region A), tumor (region B) with strong infiltration (region C) of the surrounding healthy brain tissue (region D) is clearly visible. Increased cellularity, more nuclear pleomorphism and atypia, increased proliferation (region E), microvascular hyperplasia (region F) and necrosis (region G) is displayed on the detailed section.

### Comparison [^18^F]FET and [^18^F]FAZA PET

**Fig. 4:**
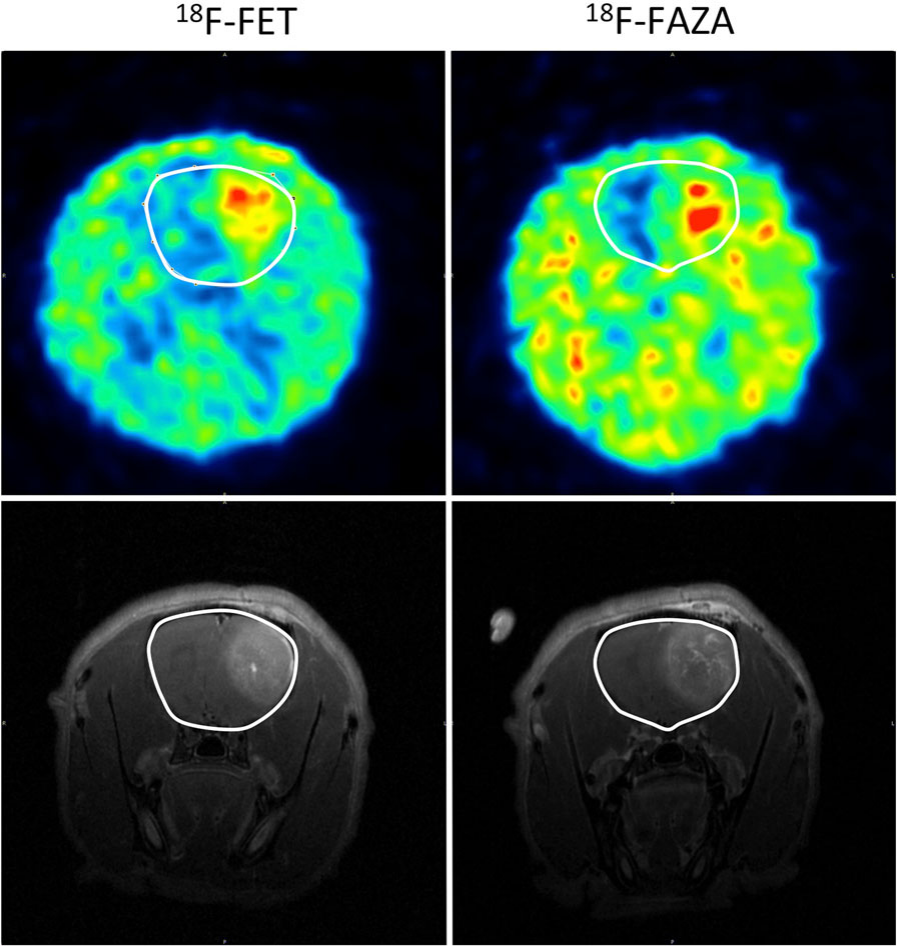
Comparison of the [^18^F]FET and [^18^F]FAZA uptake on two consecutive days in the same rat (not a rat included in the treatment groups, 13 days post inoculation). Upper row represents PET scans. Bottom row shows the contrast-enhanced T1wMRI scans. The full white line represents rat brain

The uptake of both tracers is increased in the tumor region compared to the surrounding healthy brain tissue (Fig. 4). The SUV_mean_, SUV_max_, TBR_mean_ and TBR_max_ - values of the [^18^F]FET and [^18^F]FAZA pre-RT PET scans were compared using the Mann-Whitney U test (Table 1). The average SUV_mean_ in the tumor was significantly higher for [^18^F]FET (0.80 ± 0.16) compared to [^18^F]FAZA (0.10 ± 0.05) (*p* = 0.024). A significant difference was also found for the average SUV_max_ as [^18^F]FET (1.21 ± 0.23) was significantly higher compared to [^18^F]FAZA (0.16 ± 0.07) (*p* = 0.024). The average SUV_mean_, SUV_max_ values in healthy brain tissue were also significantly higher for [^18^F]FET (SUV_mean_ 0.45 ± 0.08; SUV_max_ 0.60 ± 0.09) compared to [^18^F]FAZA (SUV_mean_ 0.07 ± 0.03; SUV_max_ 0.09 ± 0.04) (*p* = 0.024). Likewise, the average TBR_mean_ of [^18^F]FET (1.79 ± 0.33) was significantly different compared to [^18^F]FAZA (1.35 ± 0.14) (*p* = 0.048). However, between the average TBR_max_ values no significantly difference was found ([^18^F]FET: 2.72 ± 0.53; [^18^F]FAZA: 2.23 ± 0.13).

**Table 1:** SUV and TBR-values of [^18^F]FET and [^18^F]FAZA pre-RT-PET scans

		**Rat 1**	**Rat 2**	**Rat 3**	**Rat 4**	**Rat 5**	**Rat 6**	**Average**	**SD**
[^18^F]FET	SUV_mean_	0.60	0.65	1.04	0.91	0.83	0.80	0.80	0.16
	SUV_max_	0.95	1.00	1.57	1.40	1.16	1.20	1.21	0.23
	SUV_mean_ normal tissue	0.37	0.37	0.46	0.43	0.59	0.49	0.45	0.08
	SUV_max_ normal tissue	0.51	0.65	0.56	0.53	0.75	0.62	0.60	0.09
	TBR_mean_	1.60	1.73	2.24	2.13	1.40	1.64	1.79	0.33
	TBR_max_	2.56	2.68	3.38	3.26	1.97	2.46	2.72	0.53
		**Rat 7**	**Rat 8**	**Rat 9**	**Rat 10**				
[^18^F]FAZA	SUV_mean_	0.13	0.12	0.04	0.07			0.10	0.05
	SUV_max_	0.21	0.18	0.08	0.12			0.16	0.07
	SUV_mean_ normal tissue	0.10	0.08	0.03	0.05			0.07	0.04
	SUV_max_ normal tissue	0.23	0.11	0.05	0.08			0.13	0.09
	TBR_mean_	1.32	1.50	1.23	1.39			1.35	0.14
	TBR_max_	2.11	2.36	2.23	2.32			2.23	0.13

### Radiotherapy

**Fig. 5:**
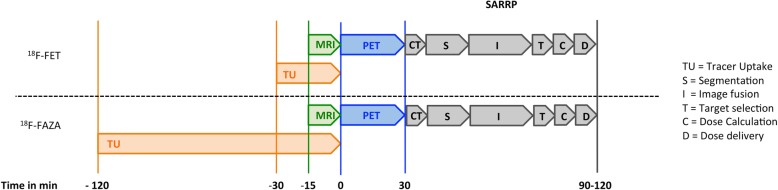
Timeline of a PET based RT experiment. The entire radiation process can take up to 4 h

MRI-based RT and PET/MRI-based sub-volume boosting using [^18^F]FET and [^18^F]FAZA were successfully performed. However, the MRI guided irradiation with PET sub-volume boosting is very time consuming. A timeline of a PET based RT experiment can be found in Fig. 5. The imaging and RT protocol can take up to 4 h.

**Fig. 6:**
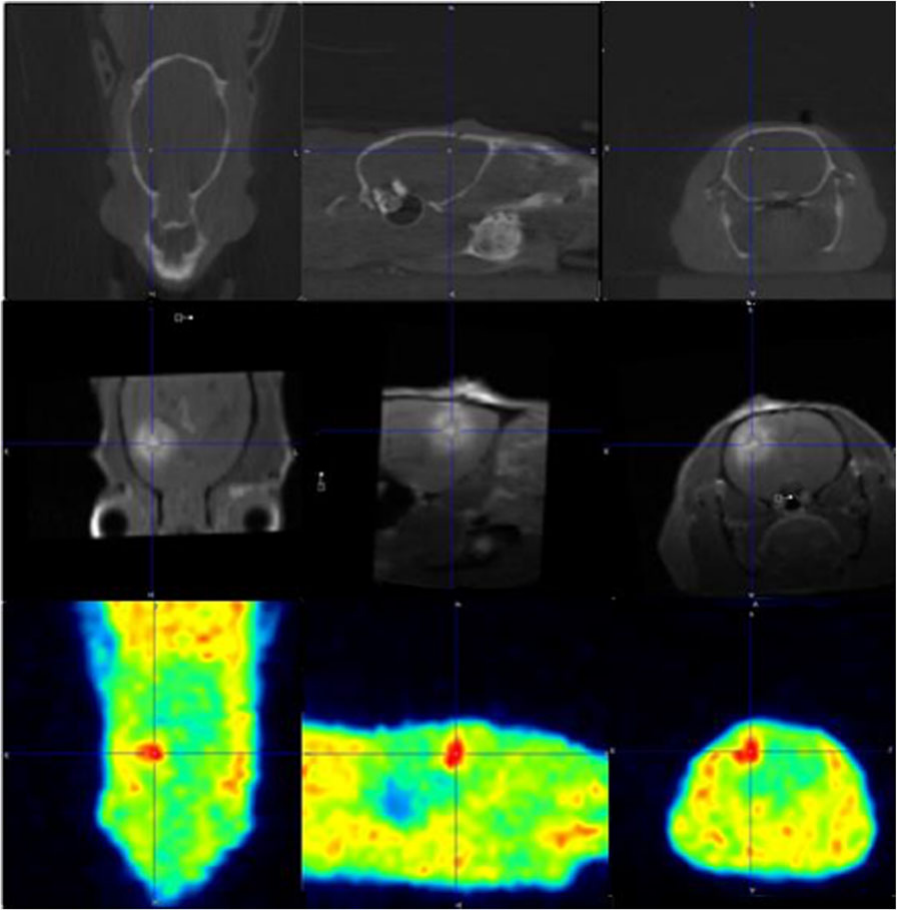
Planning CT (upper row), contrast-enhanced T1w MRI (middle row) and [^18^F]FET PET image. To perform PET-based sub-volume boosting, these three imaging modalities were co-registered

**Fig. 7:**
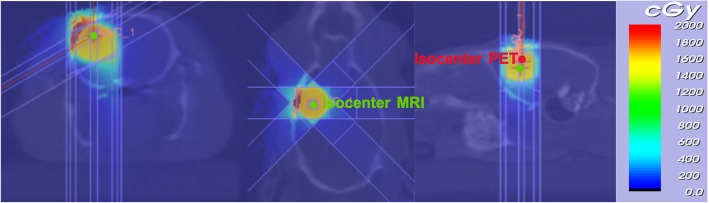
Representation of RT treatment plan using six non-coplanar arcs. One isocenter was based on the center of the contrast enhanced using a T1w MRI scan, while another isocenter was based on maximum PET tracer uptake. These two isocenters are indicated in green or red, respectively

Figure 6 shows a planning CT (upper row), a contrast-enhanced T1w MRI (middle row) and a [^18^F]FET-PET image (bottom row) that were co-registered manually into PMOD to determine the isocenters for RT planning. Figure 7 shows the dose distribution using six non-coplanar arcs as described by Bolcaen et al. [23].

### Dose volume histograms

**Fig. 8:**
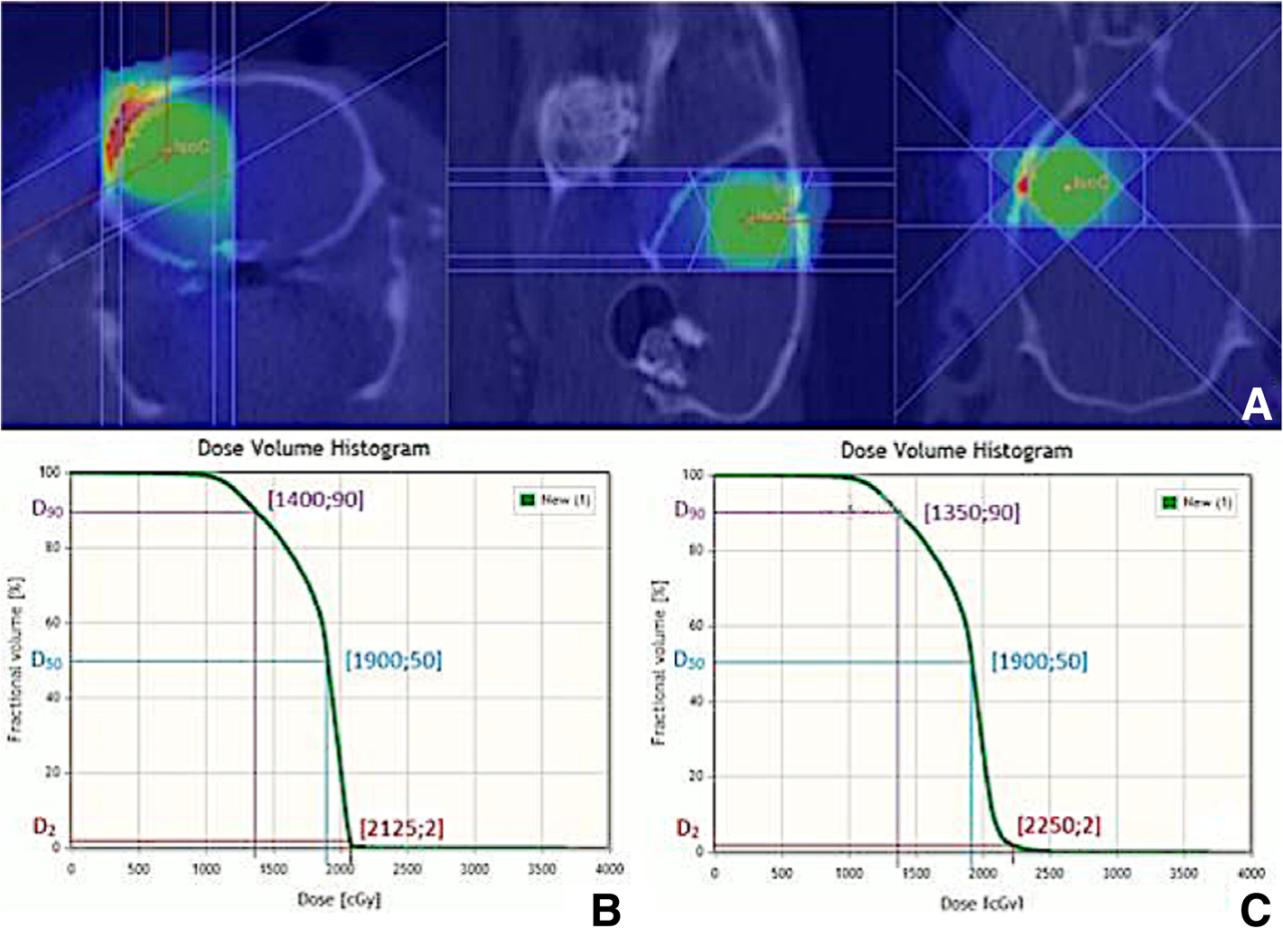
Indication of the overlapping volume of the three rotating bundles (green, **a**). Dose volume histogram with isocenter based on the center of the gadolinium containing contrast uptake on the T1wMR image (**b**). Dose volume histogram with the second isocenter based on maximum tracer uptake as shown on the PET scan (**c**)

The average, minimum and maximum dose, as well as the D_90_-, D_50_- and D_2_- values, were calculated for MRI- and PET/MRI-based RT plans (See Additional file 1: Supplementary data 1 and 2). The Mann-Whitney U test revealed that the average (1845 ± 36 cGy for MRI vs 1872 ± 36 cGy for PET), maximum (2405 ± 245 cGy for MRI vs 2560 ± 127 cGy for MRI/PET) and minimum (625 ± 146 cGy for MRI vs 654 ± 133 cGy for PET/MRI) doses were not significantly different (*p* = 0.113; *p* = 0.063; *p* = 0.489 respectively). The D_90_- (1502 ± 78 cGy for MRI vs 1513 ± 89 cGy for PET/MRI) and D_50_-values (1922 ± 34 cGy MRI vs 1933 ± 41 cGy MRI/PET) were also not significantly different (*p* = 0.666 and *p* = 0.0.546). However, the D_2_-values (2122 ± 44 cGy MRI vs 2213 ± 54 cGy PET/MRI) were significantly different (*p* = 0,002). The difference in DVH was minimal. The graph shows a slight shift to the right for the DVH based on the RT with PET/MRI based sub-volume boosting (Fig. 8). A representable DVH for the 5 Gy boost can be found in Additional file 1: Supplementary data 3.

### Response to therapy

**Fig. 9:**
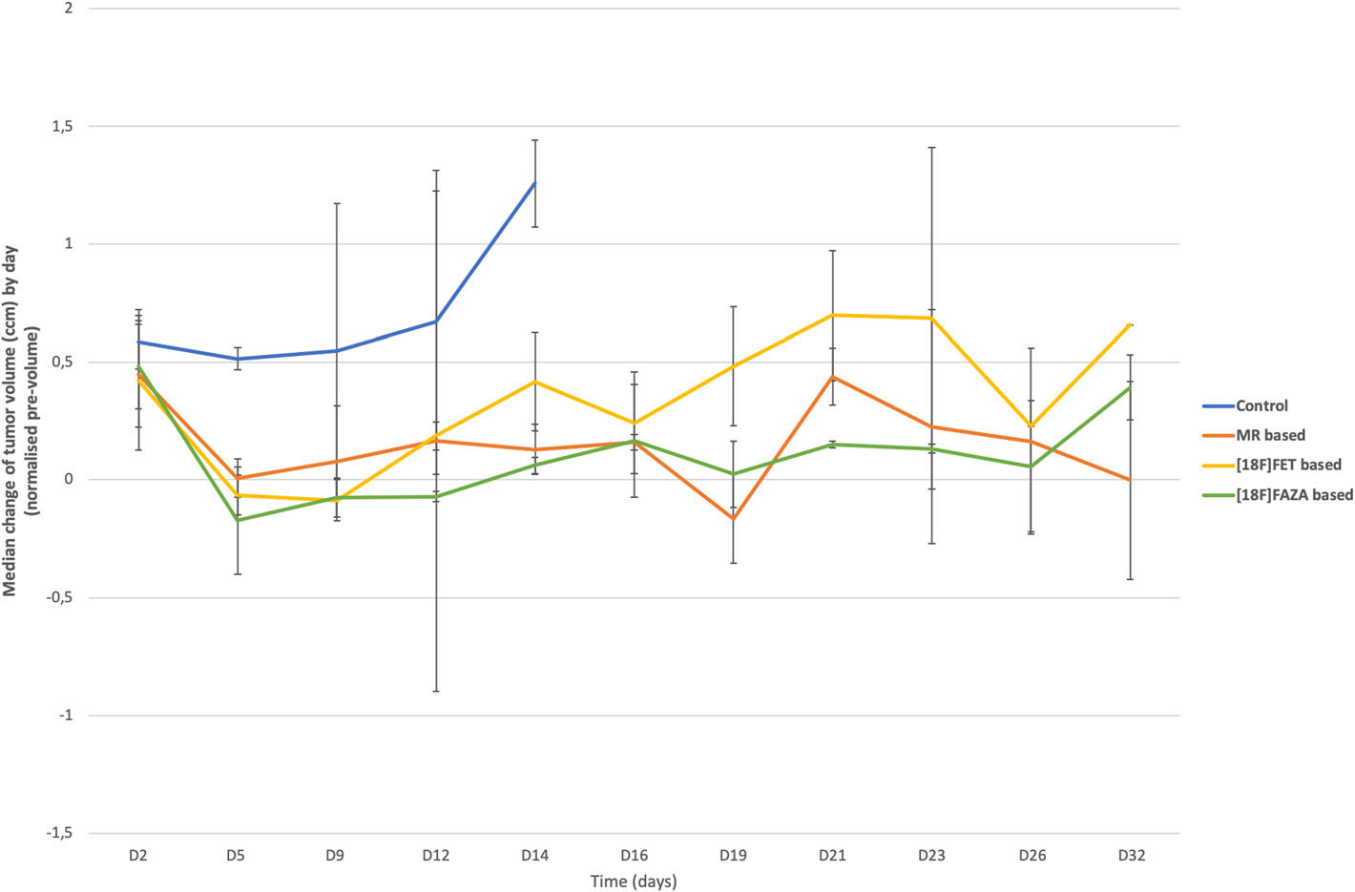
Graphical representation of the median (+SD) change of the tumor volume by day

To evaluate the response to therapy, 180 follow-up MRI scans were obtained until 36 days after tumor confirmation (Additional file 1: Supplementary data 4 and 5). Tumor volumes were delineated based on the contrast-enhancing tumor volume on T1w MRI. The absolute tumor volumes were normalized to the volume before starting RT treatment. The influence of therapy on the normalized tumor volumes is shown in Fig. 9. Tumor volumes were compared at different time points in all groups with the Mann-Whitney U test. Tumor volume on day 2 is significantly higher in the control group compared to the MRI based RT group (*p* = 0.008), while no significant difference was found compared to the PET based RT-groups ([^18^F]FET PET *p* = 0.429 and [^18^F]FAZA PET *p* = 0.413). On day 5 and 9 post therapy, the tumor volume was significantly lower for all three treatment groups compared to the control group (day 5: MRI *p* = 0.008, [^18^F]FET *p* = 0.045, [^18^F]FAZA *p* = 0.016; day 9: MRI p = 0,036, [^18^F]FET *p* = 0,024, [^18^F]FAZA p = 0,057). On day 12, a significant difference was only found between the control group and the [^18^F]FET PET group (*p* = 0.046). Compared to the [^18^F]FAZA group, the difference is borderline not significant (*p* = 0.064). After day 14 no significant differences could be found between the different therapy groups.

## Discussion

Given the poor survival of GB patients, there is an urgent need for improving current and developing new treatment methods. Multiple therapies have been proposed at the time of GB recurrence including cytotoxic chemotherapy, immunotherapy, angiogenesis inhibitors,… [30]. Unfortunately, none of these therapies turned out to be a big breakthrough [31]. Therefore, more research is being done in optimizing the currently used radiation therapy to provide the best possible outcome [18]. Although a dose-response relationship in GB is well known, escalation studies giving a higher dose to the entire tumor have not been successful [32]. PET-guided RT targeting the most metabolically active or radioresistant tumor region with RT might improve local tumor control without compromising normal surrounding brain and possibly lead to a better survival in GB patients. However, only limited studies have been performed using PET guided RT in GB rats. Menichetti et al. demonstrated the usefulness of [^18^F]FET-PET guided boron neutron capture therapy in F98 rats [33]. However, several groups have been incorporating optical molecular imaging techniques such as bioluminescence and fluorescence imaging, to guide RT successfully [34–38]. Unfortunately, optical imaging has limited spatial resolution due to tissue absorption and scatter of visible light and background artifacts need to be taken into account caused by auto-fluorescence [39]. Most importantly, bioluminescent imaging cannot be easily translated to the clinic as it requires genetic manipulation of tumor cells. As such, the implementation of PET for RT planning looks promising [40].

In our F98 GB rat model, the TBR_mean_ value of [^18^F]FET was significantly higher compared to [^18^F]FAZA, suggesting that distinction between tumor and healthy brain tissue was more evident on the [^18^F]FET PET scan.

Unfortunately, there are only few studies available that describe the uptake of [^18^F]FAZA in GB patients or in a GB rat model. In order to characterize the biological features of the F98 GB model, Belloli et al. [41] studied the uptake of [^18^F]FDG and [^18^F]FAZA. Contrary to our study, the [^18^F]FAZA uptake in the study by Belloli et al. was centrally located and showed a TBR value of 2.2 ± 0.4, whereas in our study a lower TBR_mean_ for [^18^F]FAZA, i.e. 1.35 ± 0.14, was observed. In agreement with the study by Belloli et al., visual analysis of the PET scans with different tracers showed a more centrally located [^18^F]FAZA uptake in the tumor in comparison to [^18^F]FET uptake. The latter is in agreement with the knowledge that the most hypoxic tumor regions are centrally located whereas viable tumor cells, surrounding tumor necrosis are situated in the periphery of the tumor. As described by several research groups both [^18^F]FET and [^18^F]FAZA are suitable PET tracers for the purpose of sub-volume boosting [41, 42].

In agreement with the histological findings, GB is characterized by central tumor necrosis with a viable and rapidly infiltrating tumor rim whereas hypoxic regions are observed in the middle of the tumor and regions with elevated amino acid transport are detected at the border of the tumor [19].

RT was given using three non-coplanar arcs in order to limit the dose delivered to healthy brain tissue [29, 43]. However, some shortcomings of the SARRP system should be considered. The used collimators are rigid (square aperture of 1^2^–5^2^ mm^2^) and therefore not conformed to the shape of the tumor. This might possibly be solved by developing a multi-leaf collimator as is being used in the clinic. In addition, this would also allow the use of intensity-modulated radiotherapy (IMRT), which is a common practice in the clinic. Dose painting by numbers, impossible without IMRT, was not possible due to unavailability of software needed for performing the calculations. Due to these restrictions, we opted for a set up with sub-volume boosting using a 1 × 1 mm^2^ collimator to deliver an additional dose of 5 Gy to the tumor region corresponding with the highest PET signal.

By means of DVHs the influence of the extra 5 Gy on the total tumor dose was estimated for the groups receiving the PET based subvolume boost irradiation. Only the dose that was delivered to 2% of the tumor volume (which corresponds with the maximum dose) was significantly different. This can be explained by higher dose values in the groups with PET sub-volume boosting in comparison to dose values calculated based on the MRI based irradiation. The results illustrate that there is no significant difference regarding the delivered dose located at the tumor volume between the three therapy groups. This means that the PET sub-volume boosting does not result in a higher radiation burden for the adjacent brain. It should be noted that using a 1 × 1 mm^2^ collimator to perform the boosting results in a very limited volume receiving the additional 5 Gy. If a larger target volume would be used, the DVHs would differ considerably.

Tumor volumes at different timepoints were compared with the Mann-Whitney U test. Soon after initiation of therapy, the first effect of treatment was visible showing a significant difference in tumor volumes from day 5 until day 12 between the treated and control groups. However, no significant differences were observed between the MRI based and [^18^F]FET- vs. [^18^F]FAZA-PET based RT groups. Tumor growth in the MRI based RT group corresponded to our previously published results [29], where GB was treated with RT and TMZ in the F98 rat model. The growth of the tumor remained stable until day 9 after therapy, while a continuous increase in tumor volume was observed in the control group.

Three important limitations of this study include the resolution of the PET scanner (1.2 mm) and the limited number of rats that completed the study. A more accurate delineation of the BTV might be possible by using a small animal PET scanner with better spatial resolution. Secondly, taking into account the humane endpoints, several animals had to be euthanized early due to clinical deterioration as result of large tumors as previously described by Schültke et al. [44]. This issue will influence the average values significantly. To prolong the follow-up period of the rats, inoculation of the tumor in the thigh could be an alternative [14]. However, subcutaneous glioma models lack the specific CNS microenvironment that has an essential role in glioma biology and progression [45]. Finally, the implementation of preclinical PET guided irradiation is very time consuming. A timeline is shown in Fig. 5. Using our GB rat model, we are obliged to perform the imaging and irradiation subsequently, while the rat is continuously fixed to a multimodality bed with multimodality markers fixed to the head. This is necessary to be able to perform exact manual image fusion using the radiation planning software or PMOD, as automatic image fusion is still not reliable in small animals. Patients don’t have their imaging and irradiation subsequently as image fusion software is perfectly able to fuse MRI/CT/PET images. Furthermore, manual segmentation of the CT image is necessary for dose calculation, while this is not the case in patients. Also, the preclinical RT planning has to be performed immediately after the CT to exclude a change of the rats position and to limit the total anesthesia time. In patients, the radiation therapist starts to design a RT plan after receiving the multi-modilty images and subsequently the first treatment of the patient can be initiated. As such, the timeline of the imaging and RT planning of a patient is covered over days while preclinically all these steps have to be performed in a few hours during anesthesia of the rat.

Taking the limitations into account, our data demonstrates the feasibility of dose targeting in a F98 rat GB model using MR, [^18^F]FET- and [^18^F]FAZA-PET imaging. However, technical advances are needed to apply a non-uniform dose to a target region more accurately.

## Conclusions

In this study, we showed the feasibility of PET guided subvolume boosting in a rat GB model. Comparing the effect of therapy applying MRI-based RT versus PET-based sub-volume boosting, we concluded that only a significant difference in tumor growth was found between active therapy and sham therapy respectively, while no significant differences were found when comparing the three treatment groups. Improvements for dose targeting in rodents are mandatory to apply a non-uniform dose to the more resistant regions of the tumor which might then be beneficial in terms of local tumor control. The latter combined with ne targeted drugs for GB treatment is, in our opinion, the most promising strategy.

## Additional file

Additional file 1: Supplementary data 1. Dose values for MRI based RT for the different rats. D_x_ stands for the dose that x % of the tissue volume received. Supplementary data 2. Dose values for PET/MRI-based RT with PET based sub-volume boosting. D_x_ stands for the dose that x % of the tissue volume received. Supplementary data 3. representative DVH of 5 Gy boost using a 1 × 1 mm^2^ collimator. The DVH shows that over 50% of the tissue volume received a dose of 5 Gy. Supplementary data 4. Overview of data obtained. ✓ means the scan was performed, ✕ means the scan was not obtained. Supplementary data 5. Overview tumor volumes (ccm) for all groups. The tumor volumes were based on the volumes of interest manually drawn based on the T1 weighted MRI. For the PET based volumes, a threshold of 60% maximum standardized uptake value was applied within the rat brain. Animals were euthanized when human endpoints were reached (> 20% weight loss, tumor volume > 40% of total brain volume based on MRI or signs of ataxia). (DOCX 249 kb)

## Abbreviations

BTV: Biological Target Volume
CT: Computed Tomography
CTV: Clinical Target Volume
GB: Glioblastoma
GTV: Gross Tumor Volume
HE: Hematoxylin and Eosin
ICRU-50: International Commission on Radiation Units And Measurements Report no. 50
IP: Intraperitoneal
MLEM: Maximum Likelihood Expectation Maximization
MRI: Magnetic Resonance Imaging
PET: Positron Emission Tomography
PTV: Planning Target Volume
RT: Radiation Therapy
SARRP: Small Animal Radiation Research Platform
SUV: Standardized Uptake Value
T1w: T1 weighted MRI
TBR: Tumor-to-Background Ratio
TMZ: Temozolomide
VOI: Volumes of Interest
