# Supplementary material for: Technical feasibility of [18F]FET and [18F]FAZA PET guided radiotherapy in a F98 glioblastoma rat model
Source: Radiat Oncol. 2019 May 30;14:89. doi: 10.1186/s13014-019-1290-4 (PMC6543630; doi:10.1186/s13014-019-1290-4)

**Additional file 1**

***Supplementary data 1:*** *Dose values for MRI based RT for the different rats. D_x_ stands for the dose that x % of the tissue volume received.*

|  | **Rat 1** | **Rat 2** | **Rat 3** | **Rat 4** | **Rat 5** | **Rat 6** | **Rat 7** | **Rat 8** | **Rat 9** | **Average** | **SD** |
| --- | --- | --- | --- | --- | --- | --- | --- | --- | --- | --- | --- |
| **Volume (ccm)** | 0.10 | 0.10 | 0.11 | 0.10 | 0.07 | 0.07 | 0.08 | 0.12 | 0.06 | 0.09 | 0.02 |
| **Average dose (cGy)** | 1828 | 1855 | 1833 | 1803 | 1860 | 1920 | 1857 | 1795 | 1851 | 1845 | 36 |
| **Maximum dose (cGy)** | 2171 | 2475 | 2098 | 2413 | 2866 | 2525 | 2514 | 2116 | 2470 | 2405 | 245 |
| **Minimum dose (cGy)** | 451 | 753 | 796 | 333 | 606 | 656 | 650 | 676 | 706 | 625 | 146 |
| **D90 (cGy)** | 1475 | 1600 | 1500 | 1400 | 1500 | 1600 | 1550 | 1375 | 1525 | 1502 | 78 |
| **D50 (cGy)** | 1900 | 1900 | 1925 | 1900 | 1950 | 2000 | 1900 | 1900 | 1925 | 1922 | 34 |
| **D2 (cGy)** | 2100 | 2100 | 2175 | 2125 | 2150 | 2200 | 2100 | 2075 | 2075 | 2122 | 44 |

***Supplementary data 2:*** *Dose values for PET/MRI-based RT with PET based sub-volume boosting. D_x_ stands for the dose that x % of the tissue volume received.*

|  | **Rat 1** | **Rat 2** | **Rat 3** | **Rat 4** | **Rat 5** | **Rat 6** | **Rat 7** | **Rat 8** | **Rat 9** | **Average** | **SD** |
| --- | --- | --- | --- | --- | --- | --- | --- | --- | --- | --- | --- |
| **Volume (ccm)** | 0.10 | 0.10 | 0.11 | 0.10 | 0.07 | 0.07 | 0.08 | 0.12 | 0.06 | 0.09 | 0.02 |
| **Average dose (cGy)** | 1896 | 1875 | 1856 | 1826 | 1885 | 1936 | 1883 | 1816 | 1880 | 1872 | 36 |
| **Maximum dose (cGy)** | 2645 | 2512 | 2538 | 2506 | 2867 | 2529 | 2515 | 2436 | 2490 | 2560 | 127 |
| **Minimum dose (cGy)** | 683 | 761 | 797 | 334 | 607 | 672 | 651 | 676 | 709 | 654 | 133 |
| **D90 (cGy)** | 1625 | 1550 | 1500 | 1350 | 1500 | 1600 | 1550 | 1400 | 1550 | 1513 | 89 |
| **D50 (cGy)** | 1950 | 1900 | 1025 | 1900 | 1950 | 2025 | 1950 | 1900 | 1900 | 1933 | 41 |
| **D2 (cGy)** | 2125 | 2200 | 2250 | 2250 | 2300 | 2200 | 2200 | 2150 | 2250 | 2213 | 54 |

***Supplementary data 3:*** *representative DVH of 5 Gy boost using a 1 x 1 mm^2^ collimator. The DVH shows that over 50% of the tissue volume received a dose of 5 Gy.*


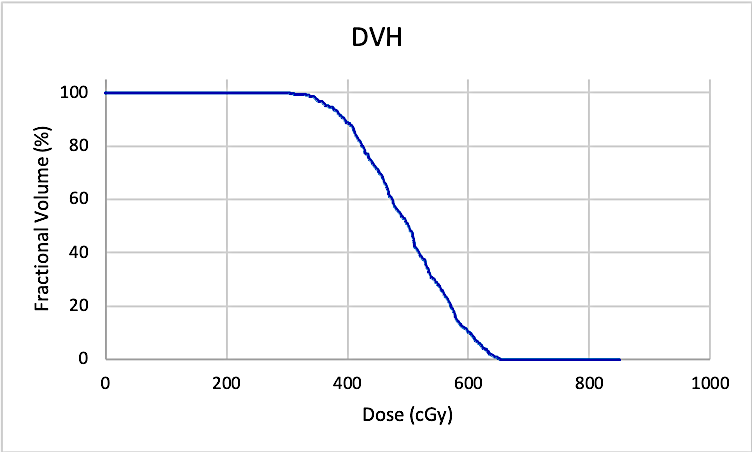


***Supplementary data 4****: Overview of data obtained.* ✓ means the scan was performed, ✕ means the scan was not obtained.


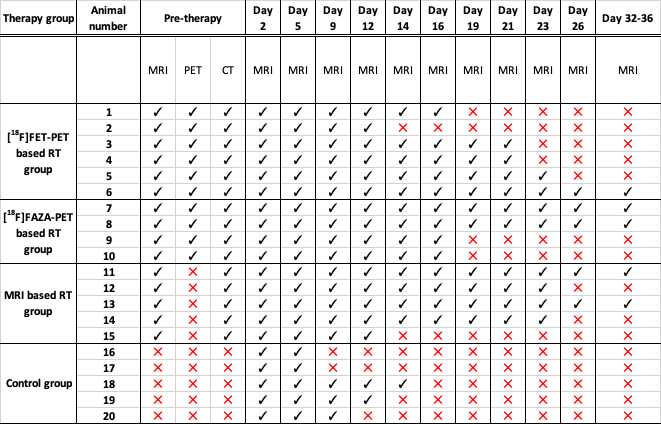


***Supplementary data 5****: Overview tumor volumes (ccm) for all groups. The tumor volumes were based on the volumes of interest manually drawn based on the T1 weighted MRI. For the PET based volumes, a threshold of 60% maximum standardized uptake value was applied within the rat brain. Animals were euthanized when human endpoints were reached (>20% weight loss, tumor volume >40% of total brain volume based on MRI or signs of ataxia).*


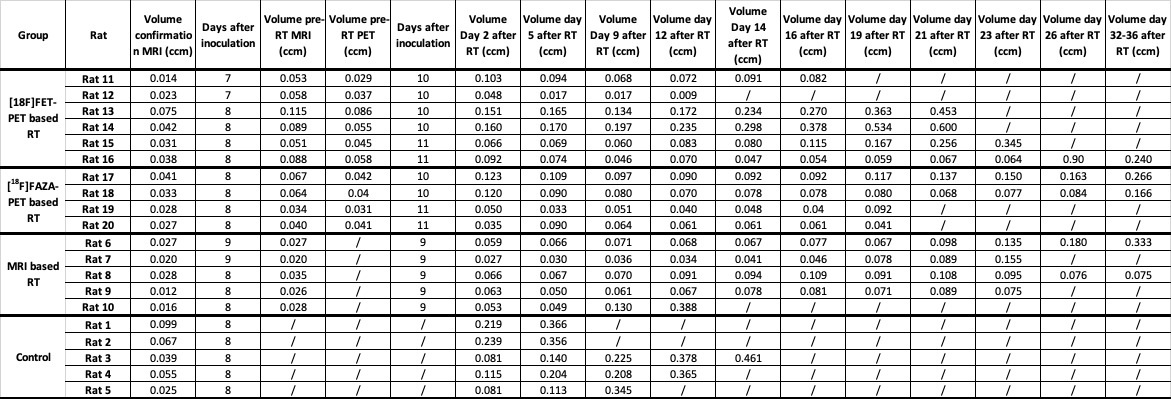

Supplement: Supplementary file 1 — Supplementary data 1. Dose values for MRI based RT for the different rats. Dx stands for the dose that x % of the tissue volume received. Supplementary data 2. Dose values for PET/MRI-based RT with PET based sub-volume boosting. Dx stands for the dose that x % of the tissue volume received. Supplementary data 3. representative DVH of 5 Gy boost using a 1 × 1 mm2 collimator. The DVH shows that over 50% of the tissue volume received a dose of 5 Gy. Supplementary data 4. Overview of data obtained. ✓ means the scan was performed, ✕ means the scan was not obtained. Supplementary data 5. Overview tumor volumes (ccm) for all groups. The tumor volumes were based on the volumes of interest manually drawn based on the T1 weighted MRI. For the PET based volumes, a threshold of 60% maximum standardized uptake value was applied within the rat brain. Animals were euthanized when human endpoints were reached (> 20% weight loss, tumor volume > 40% of total brain volume based on MRI or signs of ataxia). (DOCX 249 kb) [file 13014_2019_1290_MOESM1_ESM.docx]
